# Experimental study of thermal conductivity coefficient of GNSs-WO3/LP107160 hybrid nanofluid and development of a practical ANN modeling for estimating thermal conductivity

**DOI:** 10.1016/j.heliyon.2023.e17539

**Published:** 2023-06-22

**Authors:** Mohammad Hossein Razavi Dehkordi, As’ad Alizadeh, Hussein Zekri, Ehsan Rasti, Mohammad Javad Kholoud, Ali Abdollahi, Hamidreza Azimy

**Affiliations:** a Department of Mechanical Engineering, Najafabad Branch, Islamic Azad University, Najafabad, Iran; b Department of Civil Engineering, College of Engineering, Cihan University-Erbil, Erbil, Iraq; c College of Engineering, The American University of Kurdistan, Duhok, Kurdistan Region, Iraq; d Department of Mechanical Engineering, College of Engineering, University of Zakho, Zakho, Kurdistan Region, Iraq; e Department of Mechanical Engineering, Sarvestan Branch, Islamic Azad University, Sarvestan, Iran

**Keywords:** Thermal conductivity coefficient, Hybrid nanofluid, Graphene nanosheets, Tungsten nanoparticles, Liquid paraffin, Feed-forward neural network

## Abstract

In the present study, the effects of nanoparticles, mass fraction percentage and temperature on the conductive heat transfer coefficient of Graphene nanosheets- Tungsten oxide/Liquid paraffin 107160 hybrid nanofluid was investigated. For this purpose, four different mass fractions were used in the range of 0.005%–5% in a number of examinations. The results illustrated that the thermal conductivity coefficient was increased with the increment of the mass fraction percentage and the temperature of Graphene nanosheets- Tungsten oxide nanomaterials in the base fluid. Then, a feed-forward artificial neural network was used to model the thermal conductivity coefficient. In general, with the increase in temperature and concentration of nanofluid, the value of thermal conductivity increases. The optimum value of thermal conductivity for this experiment was observed in the volume fraction of 5% and at the temperature of 70 °C. The results of this modeling indicated that the fault of the data estimated for the coefficient of thermal conductivity in the Graphene nanosheets- Tungsten oxide/Liquid paraffin 107160 nanofluid, as a function of mass fraction percentage and temperature, was less than 3%, as compared to the experimental data.

## 1 Introduction

Nanotechnology has made it possible to produce nanoparticles small enough to be used in all physical processes [[1], [2], [3], [4], [5], [6], [7], [8]]. This technology is one of the most advanced ones today, and its various applications can change the future world. By producing nanostructures, the physical properties of materials, including the magnetic behavior, electrical conductivity, thermal conductivity, strength and melting point, can be controlled without changing the structure. These changes in properties can be made using advanced nanomaterial technology in different fields such as electrical industries, materials, chemical industries, oil and gas. Generally, nanoparticles refer to particles whose dimensions are considered less than 100 nm [[9], [10], [11], [12], [13], [14]]. Since nanoparticles are extremely small, when dispersed in liquids, they generate unique properties in those liquids, such as high thermal conductivity coefficient, longer stability period, and less erosion. Nowadays, heat exchangers are widely used in various industries such as oil, gas and petrochemical industry, power plant, car industry, etc. Since the metal solids and their oxides have a thermal conductivity much higher than that of fluids, the idea of dispersing the solid particles in the fluid to enhance this feature has arisen in the recent years. Utilization of nanofluid as a new approach is considered in the operations of heat transfer [9,10,[15], [16], [17], [18]].

First, Choi put the nanofluid idea into practice by dispersing nanometric solid particles in a fluid. However, when using these fluids, problems such as lack of stability, sedimentation, abrasion, erosion of the ducts, and pipe blockages in the used heat exchangers could prevent the acquisition of a proper commercial product on an industrial scale [19]. The first evidence on the enhancement of the conductivity coefficient of nanofluids caused by the utilization of nanoparticles in common heat transfer fluids was reported by Masuda et al. in 1993 [20]. Generally, they reported that the thermal conductivity coefficient of these fluids could be enhanced by adding small amounts of metal nanoparticles or metal oxide nanoparticles such as copper, copper oxide, alumina, or carbon nanotubes (CNT) to the fluid. On the other hand, the convective heat transfer coefficient (HTC) is the main factor in the heating-cooling applications of forced convection, and its enhancement in the nanofluid performs by parameter variations, such as the volumetric concentration of nanomaterials, size, structure of the particles' shape, temperature of the base fluid and the additives [21]. The experimental and theoretical results have shown that frequently used fluids that are being consumed in the heat transferring field have a low coefficient of thermal conductivity and thermal diffusivity coefficient. Therefore, since the conductive coefficient of nanoparticles is high due to their solid nature, the thermal conductivity coefficient of the fluid, which is one of the most fundamental parameters of heat transfer, can be increased by dispersing these nanoparticles in the base fluid [22].

Graphene-based nanofluids consist of nanoparticles; the most important ones include: Graphene (GE), Graphene Oxide (GO), Graphene nanoplatelets (GNPs), Graphene nanosheets (GNSs). These nanoparticles have been used with different aqueous and non-aqueous fluids. In some of the past studies, thermo-physical features of these GNS-based nanofluids have been investigated. Yu et al. have, for instance, investigated the thermal conductivity coefficient of the GNSs-ethylene glycol nanofluid stabilized with a conventional surfactant (sodium dodecylbenzene sulfonate (SDBS)) in the range of 0.01–0.05 wt% using the Transient Hot Wire (THW) procedure. Production of the nanofluid using the two-step method was performed and Fourier-transform infrared spectroscopy (FT-IR), Energy-dispersive X-ray spectroscopy (EDX), and Transmission electron microscopy (TEM) tests were used for the validation of the nanofluid. They demonstrated that the conductive HTC of the nanofluid along with Graphene sheets at 5 volumetric concentrations was increased by 86% [23]. Aravind et al. also investigated the thermal conductivity coefficient of GNSs-deionized water and GNSs-ethylene glycol nanofluids without any surfactant in the range of 0.008–0.138 volumetric concentrations. They produced the nanofluid by the two-step method and utilized the FT-IR, EDX and TEM tests for the validation of the nanofluid. They also demonstrated that the thermal conductivity coefficients for GNSs-deionized water and GNSs-ethylene glycol nanofluids were increased by 13.6% and 27%, respectively, at the temperature of 25 Celsius degrees [24].

As a matter of fact, hybrid nanofluids are obtained from the suspension of two different very small particles on the nanoscale in the base fluid; they are capable of increasing the heat transfer significantly, as compared to the pure fluids [25]. Some researchers have studied the conductive HTC of hybrid nanofluids along with GNSs-based nanofluids. Baby et al. have also illustrated the conductive HTC of the Ag/HEG hybrid nanofluid in the base fluid of deionized water and ethylene glycol. They produced the nanofluid by the two-step method, with a concentration of 0.01–0.07 wt%. They also used FT-IR, scanning electron microscope (SEM), X-ray Diffraction (XRD) and TEM tests for the validation of the nanofluid. They showed that the hybrid nanofluid's conductive HTC for the deionized water base fluid has was increased by 25% at 25 °C and 86% at 70 °C. They also indicated that the nanofluid's thermal conductivity coefficient for the ethylene glycol base fluid was improved by 6% at 25 °C and 14% at 70 °C [26].

According to the literature, there is no investigation on the thermophysical features of the hybrid nanofluid with GNSs [27]. It has been shown that the graphene causes the thermal conductivity coefficient to be increased [28]. On the other hand, increasing the viscosity of the nanofluid makes its application limited in industrial processes requiring high pumping power for the high rates of the heat transfer fluid [29]. Therefore, studies on increasing the efficiency of the heat transfer should be such that the viscosity of this nanofluid can be decreased. Also, it has been stated in the past studies that due to their irregular catheter movement (Brownian motion), adding spherical nanoparticles can easily lead to the separation of the intertwines of GNSs. In this case, the reduction of the intertwines of GNSs causes the momentum transfer between the layers of the fluid to be easily done. Therefore, it is expected to significantly reduce the viscosity by adding spherical nanoparticles to the nanofluid containing GNSs; meanwhile, the thermal conductivity coefficient can be kept high, so that heat transfer efficiency in the heat exchangers can be maintained; also, the nanofluid pumping costs can be reduced [30,31].

Thus, this study aimed to increase the thermal conductivity coefficient using the technology of producing hybrid nanofluids containing spherical WO_3_ nanoparticles and also, GNSs; this should be such that the viscosity of the nanofluid would not increase significantly. Since oil is being used in the heat exchangers for heat transfer, in this study, non-aqueous base fluids were considered so that an applicable conclusion in line with the existing assumptions could be achieved. Also, since the nanostructure of WO_3_ is very hydrophobic, it was expected for these nanoparticles to have relatively high stability in non-aqueous environments and a good result could be obtained. It has been determined in recent years that compared to viscosity, the thermal conductivity could be increased more with temperature, concentration, and use of nano-powders in the base fluid. To add, the nanofluids based on paraffin have a proper function in the cooling processes of high-temperature industries. In this study, the conductive HTC of the hybrid nanofluid with a non-aqueous base fluid containing GNSs and WO_3_ was investigated at different temperatures and concentrations. The use of graphene nanosheets has become widespread in all pharmaceutical, biomedical, electrical and thermal industries due to their suitable thermal, electrical and mechanical properties. In addition, WO3 is a generic wide bandgap semiconductor with a large exciton binding of energy, phonon-limited electron mobility and high optical absorption coefficient. Also, paraffin is used in all industries as a material to prevent water penetration; due to its suitable properties, this material is used as a lubricant in mechanical parts. Furthermore, this material is widely used as a phase change material in energy storage systems. Therefore, the use of these materials together can be very useful in different industries due to the unique properties of each of the components. Eventually, a proposed model for the prediction of the thermodynamic behavior of this nanofluid can be presented.

## 2 Methods and materials

In these tests, tungsten oxide nanoparticles and graphene nanosheets were dispersed in liquid paraffin. As mentioned earlier, these nanoparticles have unique and special properties and their use can increase the thermal conductivity coefficient and further improve the efficiency of thermal devices. Also, the use of an artificial neural network model, along with experimental methods, can reduce production costs and save time. Therefore, in this article, we investigated a neural network model to determine the thermal conductivity coefficient and conduct experimental tests.

### 2.1 Materials and devices

The nanoparticles formed in hybrid nanofluids are GNSs and WO_3_ nanoparticles. Due to their high conductivity, these nanoparticles have many applications in different industries. WO_3_ nano-powders, with the purity of 99.5% and physical properties presented in Table 1, were prepared from the US-Nano research Company.

**Table 1:** Characteristics of WO_3_ nano powders.

Chemical name	WO3
Molecular weight	231.8
Boiling point (°C)	1700
Melting point (°C)	1473
True density	7.16 g/cm^3^
CAS number	1314-35-8

Also, GNSs, with the purity of 99.5% and physical characteristics presented in Table 2, were prepared from the US-Nano research Company.

**Table 2:** Characteristics of GNSs.

Chemical name	Graphene nanosheets (GNSs)
Thickness (nm)	18
Number of layers	32
The diameter of Width (μm)	2–12
Special surface (m^2^/gr)	1200
CAS number	7782-42-5

Also, Liquid Paraffin 107160 was used as the non-aqueous base fluid for the production of the intended nanofluid. The liquid paraffin 107160, with the purity of approximately 99.99% and physical characteristics presented in Table 3, was obtained from the German Company Merck.

**Table 3:** The thermophysical characteristics of liquid paraffin 107160.

Flash point (°C)	300
Melting point (°C)	300–500
Vapor pressure	Less than 0.01 Pa
Kinematic viscosity	42.5 mm^2^/s
Density	0.86 g/cm^3^
CAS-No, EC-No	8012-95-1, 232-384-2

Also, devices such as TEM (9000 Na, Hitachi Co, Japan) were used to investigate the real sizes of GNSs-WO_3_ nanoparticles and the thermal conductivity coefficient; also, the Z-Potential3 (Malvern Co, England, under the ZetaSizer name) was applied to measure the surface loads of WO_3_ nanoparticles. Further, the thermal analyzer or KD-2 Pro (Deacagon Co, USA); and other experimental equipment like magnetic stirrer and erlen were used for the stabiltization of the nanofluid.

### 2.2 Preparation of the nanofluid

**Fig. 1:**
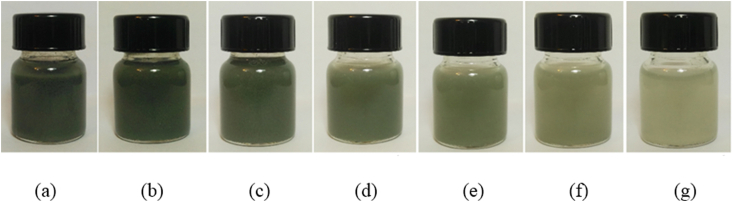
Different concentrations of hybrid nanofluids; (a) φ = 5 wt%, (b) φ = 1 wt%, (c) φ = 0.5 wt%, (d) φ = 0.1 wt%, (e) φ = 0.05 wt%, (f) φ = 0.01 wt% and (g) φ = 0.005 wt%.

In this study, the GNSs-WO_3_/LP107160 hybrid nanofluid was obtained by the two-step method. To produce and stabilize the nanofluid, first, a particular mass of GNSs and WO_3_ nanoparticles was weighed. Nanoparticles are dispersed in the fluid in the equal percentage (50-50%). Then, it was added into 19 g paraffin with high purity and a magnetic stirrer was used for 2 h at 800 rpm. Finally, the obtained hybrid nanofluid containing GNSs and WO_3_ nanoparticles was placed into ultrasonic device (for 20 min with 400 Watt power and 40 KHz frequency). As different mass concentrations should be considered and produced, the initial concentration nanofluid could be diluted and used. For example, in order to produce GNSs-WO_3_/LP107160 hybrid nanofluid with 1% mass concentration, first, 4 ml of the 5% nanofluid sample was taken by the pipette, and the intended base fluid (liquid paraffin here) was added to make the volume of 20 ml using a volumetric flask. A view of the intended hybrid nanofluids containing GNSs and WO_3_ nanoparticles is shown in Fig. 1.

### 2.3 The method of measuring the coefficient of thermal conductivity in the hybrid nanofluid

In the present study, an investigation is performed on the effectiveness of four temperatures higher than the room temperature, including 25, 40, 55, and 70 Celsius degrees, on the conductive HTC of the nanofluids. A temperature control water bath with the accuracy of 0.1 Celsius degrees was used to keep the temperature constant (for 15 min) and measure the thermal conductivity coefficient of the nanofluid. The measurement of the thermal conductivity coefficient for each nanofluid sample was performed using the THW (Transient Hot Wire) technique; it was repeated in 12 steps. The measurement period difference of each step with the next step was considered to be 5 min to keep the temperature around the measurement probe stable. In addition, Decagon KD2 Pro thermal analyzer was used to measure the thermal conductivity. This device works according to ASTM D5334-08 and IEEE 442-1981 standards. The probe used to measure the thermal conductivity is the KS-1 probe. Also, the measurement accuracy of the device is equal to 0.001 w/m.K. In this study, to measure the conductive HTC of the nanofluid accurately and avoid the effects of the experiment faults, first, the nanofluid containing GNSs-WO_3_ nanoparticles with different concentrations (from 0.005% to 5% mass concentration) was poured into a cylindrical glass container, such that the height of the nanofluid inside the container was higher than 10 cm (the geometry of the glass container was considered such that the height of the container was 12 cm and its diameter was 5 mm). Then, the probe (the thin rod for measuring the conductive HTC) of the measuring device was taken into the nanofluid and the value of the coefficient of thermal conductivity in the nanofluid was measured using the device. After measuring the coefficient of thermal conductivity in the nanofluid using the thermal analyzer device, the glass container was emptied and a nanofluid with a higher concentration was transferred into the container, so that the interfering effects of the nanoparticle concentrations on the final results of the test could be avoided. In this step, as in the previous ones, the device probe was immersed into the nanofluid and the coefficient of thermal conductivity was measured.

## 3 Results and discussion

### 3.1 The transmission electron microscopy (TEM) test

**Fig. 2:**
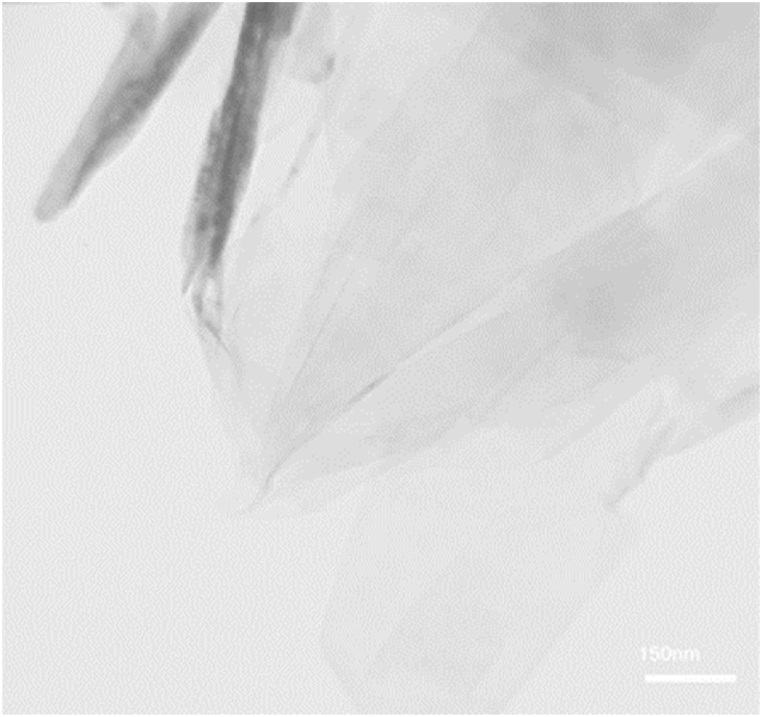
The outcomes of the TEM test for the GNSs with sheet structures, with a with a 150 nm magnification.

**Fig. 3:**
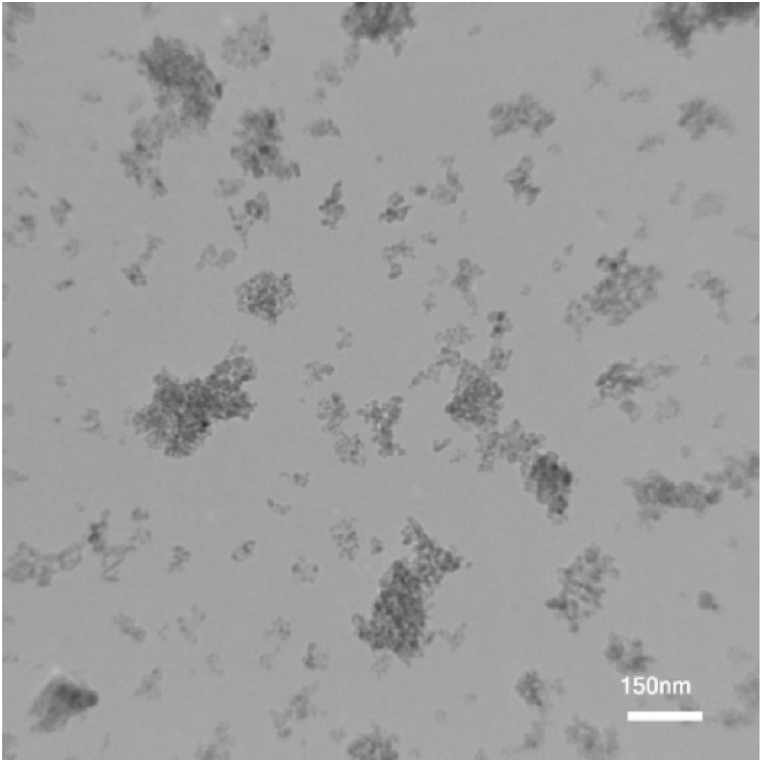
The outcomes of the TEM test for the WO_3_ nano-powders with spherical structures, with a 150 nm magnification.

In the present study, the TEM test was utilized to specify geometry, morphology and shape of GNSs-WO_3_ nanoparticles. In this test, WO_3_ nanoparticles or GNSs were added to a solvent with high volatility, and around 0.1 cc of the solution, after sufficient dilution, was transferred on a graphite sheet. After the used volatile solvent was completely evaporated, the TEM test was done on the graphite surface containing the WO_3_ nanoparticles or GNSs. Fig. 2, Fig. 3 depicts the results of the TEM images of the GNS with sheet structures and WO_3_ nanoparticles with spherical structures.

As can be seen, the geometry, shape and also, the average dimensions of the nano-powders could be specified. In this study, the shape of the WO_3_ nanoparticles were approximately spherical.

### 3.2 The zeta potential test

**Fig. 4:**
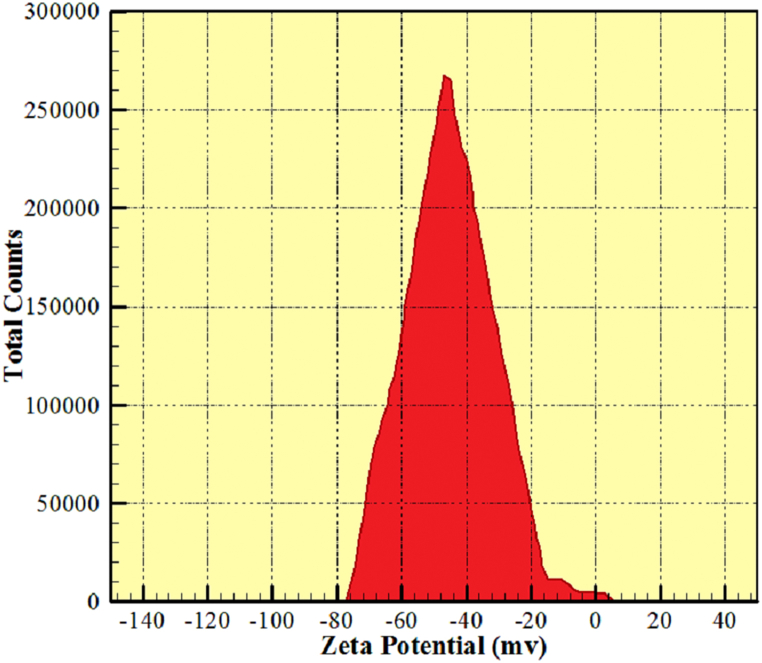
The outcomes of the Zeta Potential test of the hybrid nanofluid.

In order to study the GNSs-WO_3_ nanofluid stability in the liquid paraffin 107160 base fluid, given the lack of sedimentation of the nano-powders, one of the proper criteria is measuring the electrical surface charges of the particles dispersed in the intended base fluid. The high electrical surface charges of both negative and positive particles could lead to the stability of particles in liquids and their non-settlement. This has been very much considered in the field of nanoparticles to prevent their agglomeration. Also, the electrical surface charges could prevent the particles from being agglomerated in the liquid paraffin base fluid. In the present study, the Zeta Potential tests with high accuracy were utilized to evaluate the nanofluid stability. Most liquids contain cations and anions (positively and negatively charged atoms). The stability of nanofluids is determined according to the DLVO theory and based on the balance between attractive and repulsive forces. This theory is a classical explanation to express the stability of colloids in a suspension. It seems, therefore, that the balance between the opposing forces of electrostatic attraction and repulsion is used to explain the occurrence or non-occurrence of agglomeration of nanofluids. Also, due to the nature of these particles and the base fluid, the criterion of the stability of the particles in the fluid is that the highest surface charges should be less than −40 or higher than +40 mV. Therefore, considering that the highest peak observed in Fig. 4 is greater than 40, it could be concluded that this nanofluid has a very good stability. Therefore, the possibility of forming agglomerate in this fluid is very low. This figure depicts the outcomes of the Zeta Potential test for the hybrid nanofluid after the dispersion of the nanoparticles, sonication (20 min' ultrasound for dispersion), and preparation of the nanofluids. Also, the measurement was done one week after making the samples. Considering that the obtained results were in a suitable range, we realized that the samples have good stability. The results, as shown in the diagrams of the Zeta Potential test, indicated that the nanoparticles had very high stability in the liquid paraffin 107160 base fluid. Therefore, because of the nanofluid stability, the sedimentations of the nano-powders and its effectiveness on the physical characteristics of the nanofluid, including the thermal conductivity coefficient, could be ignored.

### 3.3 The results of investigation of thermal conductivity coefficient of the GNSs-WO_3_/LP107160 nanofluid

Many experimental and numerical studies [[32], [33], [34], [35]] have been previously performed on the use of nanomaterials to increase heat transfer. The main factor in increasing heat transfer has been the thermal conductivity of nanoparticles [[36], [37], [38], [39], [40]]. Examination of these sources showed that there was a good agreement between the trend of changes in thermal conductivity with the previous results.

**Fig. 5:**
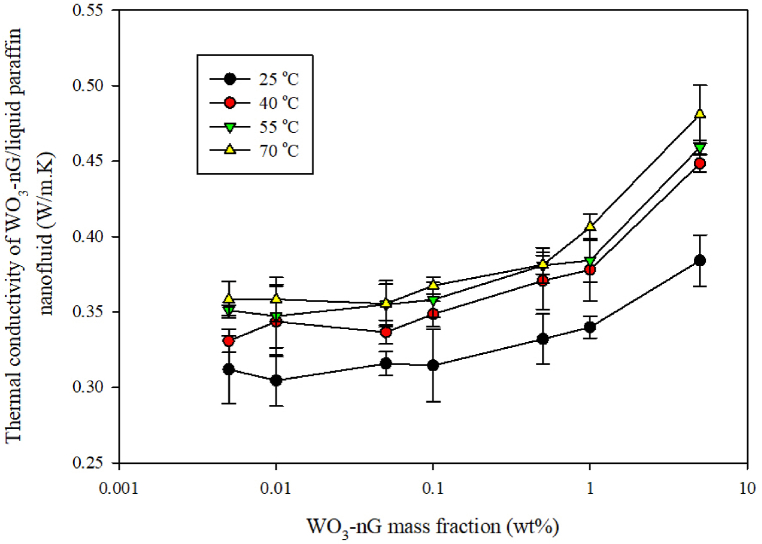
The diagram of the thermal conductivity coefficient of the nanofluid in terms of mass fraction of the GNSs-WO_3_ nanoparticles for the hybrid nanofluid at different temperatures.

After the preparation of the GNSs-WO_3_/LP107160 hybrid nanofluid, the tests were performed to measure the thermal conductivity coefficient and consider the effectiveness of changes in the mass concentration and temperature of the GNSs-WO_3_ nanopowders on the coefficient of thermal conductivity. In order to study the effectiveness of the nanopowders concentration, the GNSs-WO_3_/LP107160 hybrid nanofluid was obtained in mass concentrations ranging from 0.005% to 5%. Also, in order to investigate the effect of temperature on the thermal conductivity coefficient of the nanofluid, different temperatures, including 25, 40, 55 and 70 Celsius degrees, were selected. Fig. 5 displays the results of measuring the conductive HTC of the hybrid nanofluid of GNSs-WO_3_/LP107160 at diverse temperatures and GNSs-WO_3_ nanopowders mass fractions. It should be noted that in this diagram, the test fault was calculated using the data standard perversion from the average values.

Also according to Fig. 5, as a physical point of view, it can be concluded that the Brownian motion of nanoparticles increases with increasing temperature. Further, with the increase of these movements, the thermal conductivity coefficient increases.

Based on the results in this diagram, it could be derived that the rise of the mass concentrations of the GNSs-WO_3_ nano-powders led to the enhancement of the coefficient of thermal conductivity in the hybrid nanofluid (GNSs-WO_3_/LP107160). Also, these results demonstrated that while the temperature increase reduced the thermal conductivity coefficient of the liquid paraffin 107160 (without the nanoparticles), for this hybrid nanofluid, the rise of temperature leads to the improvement of the conductive HTC. As shown in this diagram, at 70 °C, the thermal conductivity coefficient was increased with nanoparticle concentrations; also, by increasing the temperature, the effects of temperature changes on the conductive heat transfer coefficient of the GNSs-WO_3_/LP107160 nanofluid was reduced significantly.

### 3.4 The results of relative thermal conductivity (RTC) coefficient in GNSs-WO_3_/LP107160 hybrid nanofluid

Generally, the RTC coefficient of the mentioned hybrid nanofluid could be considered as a subordinate of the nanofluid thermal conductivity coefficient divided by the thermal conductivity coefficient of the liquid paraffin 107160, as represented below [34]:

(1) $$R_{k-nf}=\frac{k_{nf}}{k_{bf}}$$

**Fig. 6:**
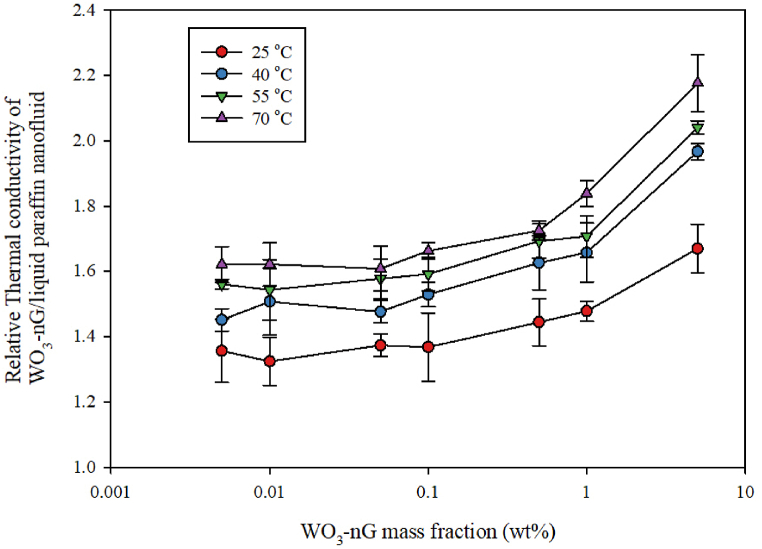
The diagram of the RTC coefficient of the hybrid nanofluid in terms of mass concentration for the GNSs-WO_3_/liquid paraffin 107160 hybrid nanofluid at varied temperatures.

In this formula, we measured K_nf_ at the given temperature and nanoparticle concentration and K_bf_ at the given temperature. Fig. 6 display the RTC coefficient of the GNSs-WO_3_/LP107160 hybrid nanofluid at varied mass concentrations of GNSs-WO_3_ nanoparticles and temperatures. The figure illustrates that by increasing the GNSs-WO_3_ nanoparticles fractions in the nanofluid with paraffin in the liquid phase as its base fluid, the changes of the RTC coefficient of the nanofluid in concentrations less than 0.1% could be very minimal. However, by increasing the mass concentrations of the nano-powders from 0.1 to 5%, the variations in the RTC of the nanofluid would be much more. Also according to Fig. 6, It can be concluded that by increasing the volume fraction of nanofluid, the active surface of heat transfer increases. Therefore, the values of thermal conductivity coefficient and relative thermal conductivity coefficient increase with increasing temperature.

**Fig. 7:**
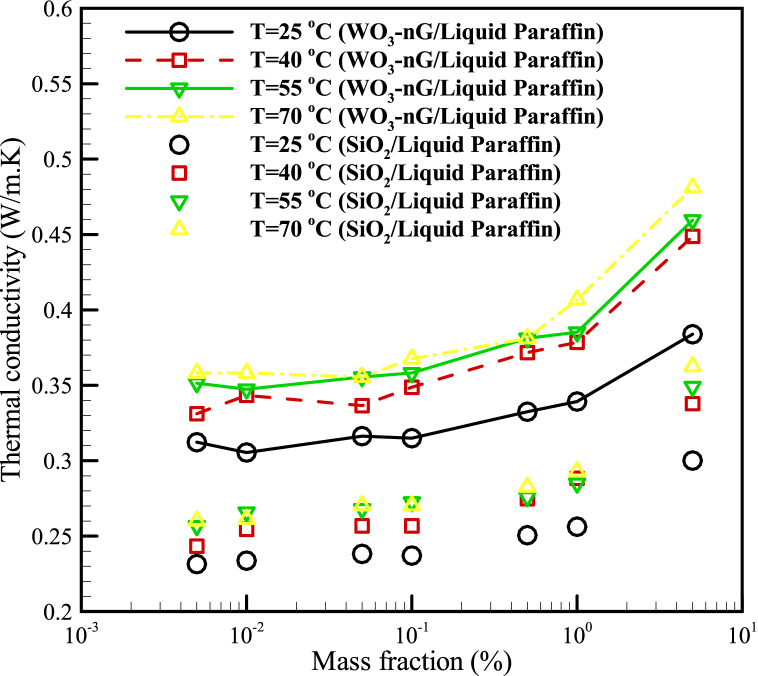
Comparing the thermal conductivity of the WO_3_-nG/Liquid Paraffin nanofluid (present work) and thermal conductivity of SiO_2_/Liquid Paraffin nanofluid (Li et al. [17]): Experimental results.

By comparing the results of this study with previous research [17], it was found that due to the use of hybrid nanoparticles (nano graphene (nG) and WO_3_), a higher thermal conductivity coefficient can be achieved. In general, using the hybrid nanoparticles (oxide nanoparticles such as WO_3_, TiO_2_, etc., and other nanoparticles such as nG, MWCNT, etc., with low and high thermal conductivities, respectively) in compare of using only oxide nanoparticles, caused to a significantly increase in the thermal conductivity coefficient and as a result thermal efficiency of the processes. For example, as seen in Fig. 7, at a temperature of 25 °C and a volume fraction of 0.005%, the thermal conductivity coefficient can be increased to approximately 35% by using hybrid nanoparticles. It can also be concluded that due to the economic point of view, the use of graphene nanoparticles is a bit expensive, the use of tungsten oxide particles along with graphene can increase efficiency and thermal conductivity in addition to reducing costs. Also, due to not using oleic acid in this study, we can achieve a stable nanofluid. According to Fig. 7 and considering the bonds made between nanoparticles and the base fluid, as well as the appropriate increase in the thermal conductivity coefficient, the use of this nanofluid is recommended.

Since it is very expensive and difficult to perform the test for all volume fractions of nanofluid, using the obtained equations in Table 4 can lead to saving the cost and time of performing the test. So, in Table 4, the equations of the thermal conductivity coefficient are presented in terms of temperature changes and volume fraction. Using of these equations, a good approximation of the behavior of the thermal conductivity coefficient can be obtained in terms of other variables.

**Table 4:** Equations for thermal conductivity coefficient in terms of temperature and volume fraction variations.

φ (%)	Equations
**0.005**	K = −0.00000348T^3^ + 0.00044873T^2^ − 0.01137678T + 1.40972296
**0.01**	K = 0.00000800T^3^ − 0.00125724T^2^ + 0.06766093T + 0.29250543
**0.05**	K = −0.00000226T^3^ + 0.00025559T^2^ − 0.00251641T + 1.32158580
**0.1**	K = 0.00000678T^3^ − 0.00099356T^2^ + 0.05280863T + 0.57087975
**0.5**	K = 0.00001322T^3^ − 0.00197708T^2^ + 0.09786293T + 0.02909358
**1**	K = 0.00001113T^3^ − 0.00165629T^2^ + 0.08444411T + 0.22483889
**5**	K = 0.00001513T^3^ − 0.00233185T^2^ + 0.12296930T − 0.18489617

### 3.5 The proposed model for the nanofluid thermal conductivity coefficient

Many numerical and analytical methods were used to modeling and simulation the industrial applications [[41], [42], [43], [44], [45], [46], [47], [48]]. In the following, the conductive HTC of the GNSs-WO_3_/LP107160 hybrid nanofluid was considered as a function of temperature and mass concentrations of the nanopowders in the base fluid. To that end, ANN was performed to solve the fitting problem between the inputs (i.e., mass concentrations and temperature) and targets (i.e., the thermal conductivity coefficient of the hybrid nanofluid). The used ANN is a feed-forward network with one hidden layer and an output layer in which “tansig” and “purelin” activations functions are used, respectively.

**Fig. 8:**
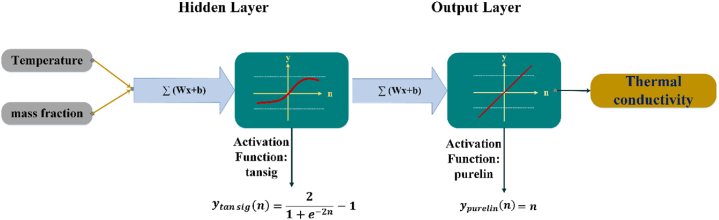
The architecture of artificial neural network.

The neural network provides the mapping relationships, as follows [[49], [50], [51]]:

(2) $$y=purelin\left(LW^{2,1}a^{1}+b^{2}\right)$$

where

(3) $$a^{1}=tansig\left(IW^{1,1}x+b^{1}\right)$$

where, x and y are the input and target vectors, $IW^{1,1}$ and $LW^{2,1}$ are the weight matrices, and $b^{1}$ and $b^{2}$ are the bias vectors of the hidden and output layers, respectively. Fig. 8 shows a view of the current network architecture. To obtain the weight and bias values, the Bayesian regularization algorithm is utilized. The sigmoid layer and linear output layer in the error propagation network's architecture enable it to estimate any function with limited points of discontinuity.

In artificial neural networks, one of the practical methods of obtaining optimal parameters is the use of Bayesian regularization learning rules, which automatically sets appropriate values for the parameters of the objective function. The Bayesian regularization algorithm can be used to improve the ability of the neural network and to train the objective function F, which is shown in Eq. (4) [[49], [50], [51]]:

(4) $$F=\alpha \frac{1}{n}\sum_{i=1}^{n}{\left(t_{i}-o_{i}\right)}^{2}+\beta \frac{1}{n}\sum_{i=1}^{n}W_{i}^{2}$$

Here, n is the number of network inputs, $t_{i}$ is the target and $o_{i}$ is the output of the ANN. In Eq. (4), the first term is the sum of the squares remaining between the network output and the objective function, and the second term is the sum of the squares of the network weights. α and β are the parameters of the objective function setting, each of which depends on the training of the network in reducing the remaining outputs or the size of the network.

In multilayer feedforward networks, neurons with a nonlinear transfer function and a linear final layer provide the ability to learn between inputs and outputs. The optimal number of neurons in the hidden layer in ANN is determined by trial and error. In this study, the number of neurons was changed between 2 and 10, and the mean relative errors was investigated according to Eq. (5). Here, n, $y_{i}$ and ${\bar{y}}_{i}$ are the number of data, target and network output, respectively. As can be seen in Table 5, the lowest mean relative error was obtained with 5 neurons in the hidden layer. The results, as can be seen in Table 5, showed that the increase in the number of neurons caused overfitting because the network could not generalize the patterns in the training data set to the test data set [[49], [50], [51]].

(5) $$Mean\;Relative\;Error=\frac{1}{n}\sum_{i}^{n}\frac{\left|y_{i}-{\bar{y}}_{i}\right|}{y_{i}}$$

**Table 5:** Mean relative error value to estimate the number of neurons in the hidden layer.

Neuron	Mean relative error %
2	4.56
3	3.12
4	2.98
5	2.82
6	3.77
7	4.46
8	4.20
9	5.14
10	6.12

**Fig. 9:**
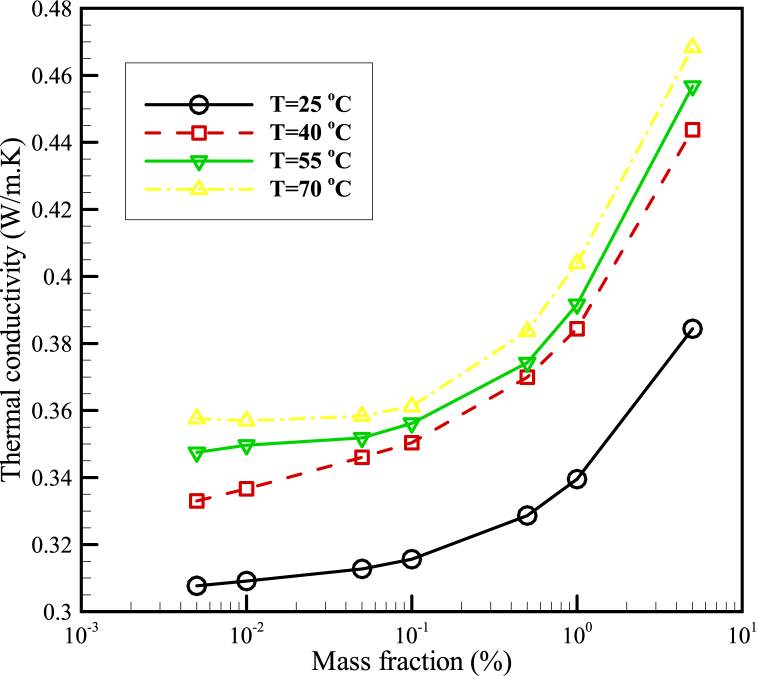
The output results of the neural network model for the tested values.

**Fig. 10:**
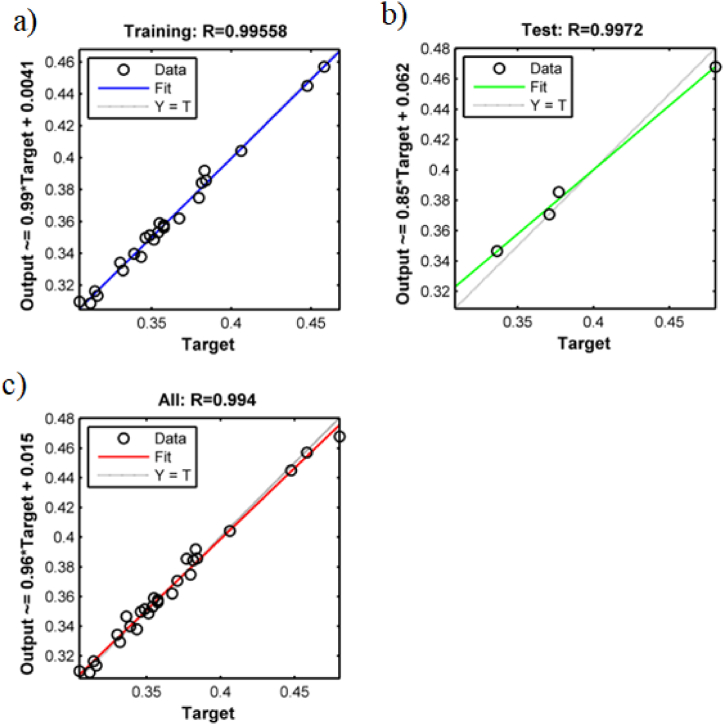
Network regression graph for a) training data, b) test data and c) all data.

**Fig. 11:**
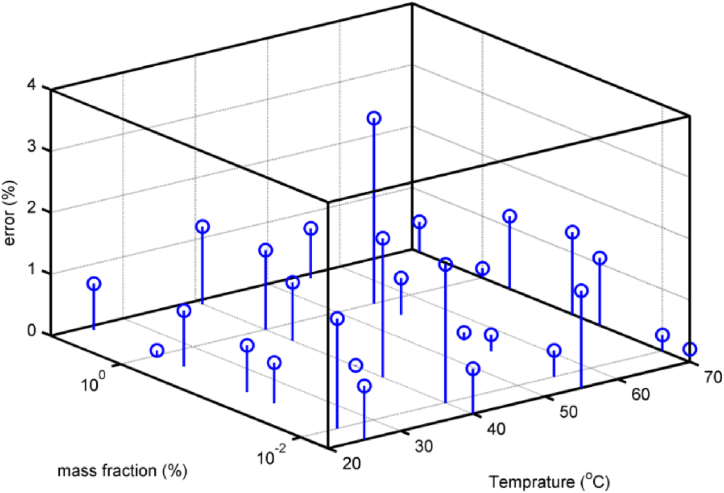
The error of the estimated thermal conductivity coefficient as a function of temperature and mass concentration, in comparison with the empirical data.

The output results of the neural network model for the tested values are shown in Fig. 9. As can be seen, the conductive heat transfer coefficient values at different temperatures for untrained data were determined using an artificial neural network in terms of mass fraction. One of the advantages of training ANN using the Bayesian regularization algorithm is that it divides the input data into sets of training and testing data. This factor causes more data to participate in the training and testing phase, and the network has a better generalization ability for the arbitrary input data. In this study, 85% of the data was considered for the training dataset and 15% for the test dataset. The regression diagram and the whole data are presented in Fig. 10 for training and testing. These graphs show how close the output data is to the actual target values in the training and test phase. As can be seen in the figures, the horizontal axis is the experimental data and the vertical axis is the outputs from ANN. In accordance with the regression diagram for the test data, which serves as a means of data validation. Values at the target and output have strong convergence. And this shows that data was correctly trained, as well as validating untrained data. In Fig. 10, the R correlation shows the closeness of the outputs obtained from the network outputs and the real values, whose value is obtained from Eq. (6) [51]. As can be seen, the network has a good correlation with the targets for the test and training dataset. The value of R = 0.994 for all data confirmed that the obtained fitting was almost ideal. Furthermore, the estimated thermal conductivity coefficient error of the GNSs-WO_3_/LP107160 nanofluid is given in Fig. 11 as a function of temperature and mass concentration, in comparison with the experimental data.

(6) $$R=\frac{n\left(\sum y_{i}{\bar{y}}_{i}\right)-\left(\sum y_{i}\right)\left(\sum {\bar{y}}_{i}\right)}{\sqrt{\left[n\sum {y_{i}}^{2}-{\left(\sum y_{i}\right)}^{2}\right]\left[n\sum {{\bar{y}}_{i}}^{2}-{\left(\sum {\bar{y}}_{i}\right)}^{2}\right]}}$$

**Fig. 12:**
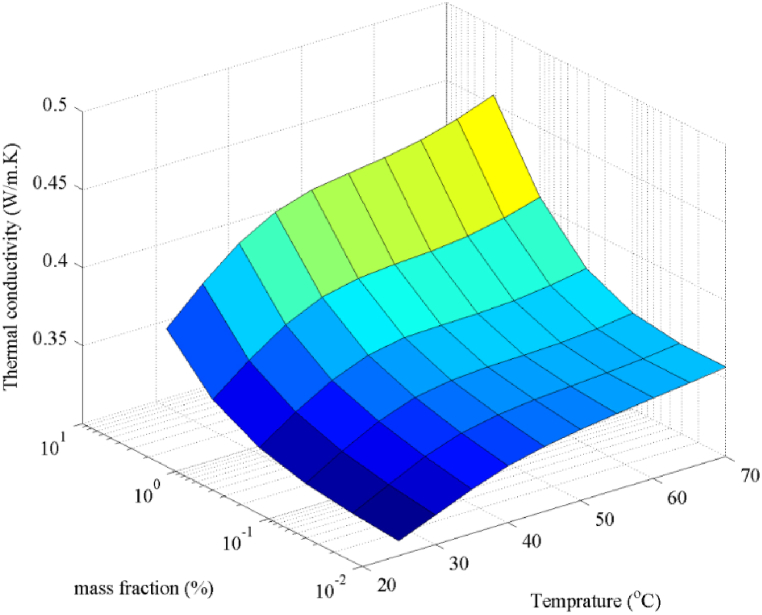
The estimated thermal conductivity coefficient in all ranges of the mass concentration and temperature inputs.

As can be seen, this error is less than 3%. When the model with proper accuracy was achieved, it can be used to estimate the coefficient of thermal conductivity in all ranges of temperature and mass concentration inputs. The final results are shown in Fig. 12. The smooth figure of this contour represents the fact that not only does the model have a proper precision for the trained data, but it also has an acceptable generalization for the untrained data as well. Therefore, this model can be used in the range of the presented inputs. As observed in the figure, the conduction heat transfer coefficient considerably rises with temperature and mass fraction, reaching a value of approximately 0.47 w/m.K at a mass fraction of 5% and a temperature of 70 °C.

## 4 Conclusion

In the present investigation, the conductive HTC of the GNSs-WO_3_/LP107160 nanofluid was studied. In order to examine the effectiveness of mass fractions and temperature of the nano-powders on the thermal conductivity coefficient of the nanofluid, four different mass fractions and temperatures of GNSs-WO_3_ nanomaterials in the range of 0.005%–5% were used in the experiments. In this study, tungsten oxide-graphene nanosheets/liquid paraffin hybrid nanofluid was used for the first time. The use of this nanofluid provided amazing results due to the unique properties of nanoparticles and base fluid. The main novelty of this research was to measure the thermal conductivity coefficient of the hybrid nanofluid. According to the results, it can be concluded that the use of this nanofluid is justified due to its properties. Also, the use of an artificial neural network model along with the experimental method helped the quality of the paper. The results of this research include:

• The conductive HTC of the nanofluid containing GNSs-WO_3_ nanoparticles changes with temperature, such that it strongly a function of temperature. The difference is that the coefficient of thermal conductivity of the liquid paraffin 107160 reduced by increasing the temperature.
• The outcomes of the present study indicated that the thermal conductivity coefficient of the nanofluid is enhanced by incrementing the mass concentrations of GNSs-WO_3_ nano-powders in the base fluid.
• The outcomes of the present study demonstrated that by incrementing the concentrations of GNSs-WO_3_ nano-powders in the nanofluid with the liquid paraffin as its base fluid, the changes of the RTC coefficient of the nanofluid in mass concentrations less than 0.1% is very little. However, by incrementing the mass concentrations of the nano-powders from 0.1 to 5%, the variations in the RTC coefficient of the nanofluid will be too larger.
• A model, as a function of mass fraction and temperature, was proposed for estimation of the conductive HTC of the GNSs-WO_3_/LP107160 hybrid nanofluid. This model not only has a proper precision for the trained data, but it also has an acceptable generalization for untrained data as well. Therefore, this model can be used in the ranges of the presented inputs.

In this study, we do not know the surface tension and density of the hybrid Nanofluids. So, in future works, we will try to investigate surface tension and the effect of density on heat transfer.

## Future extensions

For future research activities, we expect that the effect of paraffin's with different melting and freezing temperatures will be investigated. Also, investigating the Brownian motions of nanoparticles in paraffin during melting and freezing can be an interesting subject.

## Author contribution statement

Mohammad Hossein Razavi Dehkordi: Conceived and designed the experiments; Analyzed and interpreted the data; Wrote the paper.

As’ad Alizadeh: Performed the experiments; Contributed reagents, materials, analysis tools or data.

Hussein Zekri: Performed the experiments; Wrote the paper.

Ehsan Rasti: Analyzed and interpreted the data; Wrote the paper.

Mohammad Javad Kholoud: Contributed reagents, materials, analysis tools or data; Wrote the paper.

Ali Abdollahi: Contributed reagents, materials, analysis tools or data.

Hamidreza Azimy: Conceived and designed the experiments; Wrote the paper.

## Data availability statement

The authors do not have permission to share data.

## Declaration of competing interest

The authors declare that they have no known competing financial interests or personal relationships that could have appeared to influence the work reported in this paper.

## Nomenclature

k: Thermal conductivity coefficient, W/m.K
T: Temperature, °C
L: Length, cm
nm: Nanometer
E: Error
R: Regression
RTC: Relative Thermal Conductivity
gr: Gram, Mass
GNSs: Graphene Nanosheets
WO_3_: Tungsten Oxide
Pa: Pressure, Pascal
LP: Liquid Paraffin
TEM: Transmission Electron Microscopy
SEM: Scanning Electron Microscope

### Greek symbols

*ν*: Kinematic viscosity, mm^2^/s
***ρ***: Density, kg/m^3^
φ: Mass fraction, %

### Subscripts

nf: Nanofluid
bf: Base fluid
w: Water
